# Probiotic intervention improves gingival status and influences the salivary levels of Matrix Metalloproteinase (MMP)-9 and Tissue Inhibitor of metalloproteinases (TIMP)-1 in healthy adults

**DOI:** 10.1080/20002297.2017.1325214

**Published:** 2017-06-01

**Authors:** Heli Jäsberg

**Affiliations:** ^a^ Department of Cariology and Restorative Dentistry, Institute of Dentistry and FINDOS-Turku (Finnish Doctoral Program in Oral Sciences), University of Turku, Turku, Finland

## Abstract

The effect of orally administered *Bifidobacterium animalis* subsp. *lactis* BB-12 and *Lactobacillus rhamnosus* GG on the amount of plaque, gingival inflammation, oral microbiota and salivary mutans streptococci, as well as on levels of Matrix Metalloproteinases (MMP)-8, MMP-9, and of Tissue Inhibitor of Metalloproteinases (TIMP)-1 in healthy adults was studied.

The study was a randomized controlled trial with a four-week study period. The saliva samples were collected and clinical parameters measured at the baseline and at the end of the study.

In the probiotic group, the plaque and gingival indices reduced, salivary MMP-9 levels increased (p=0.007) and TIMP-1 levels decreased (p=0.003) significantly during the intervention. These changes were not observed in control group. In addition, the probiotic intervention had no effect on salivary microbiota.

To conclude, improved gingival status with increased MMP-9 and decreased TIMP-1 levels in saliva may indicate that probiotics have immunomodulatory effects in the oral cavity.

